# Laser ablation‐inductively coupled plasma‐mass spectrometry analysis reveals differences in chemotherapeutic drug distribution in surgically resected pleural mesothelioma

**DOI:** 10.1111/bcp.15813

**Published:** 2023-07-14

**Authors:** Anna Tisza, Thomas Klikovits, Michal Benej, Szilvia Torok, Beata Szeitz, Zsuzsanna Valko, Mir Alireza Hoda, Balazs Hegedus, Maximilian Bonta, Winfried Nischkauer, Konrad Hoetzenecker, Andreas Limbeck, Karin Schelch, Viktoria Laszlo, Zsolt Megyesfalvi, Balazs Dome

**Affiliations:** ^1^ Department of Tumor Biology National Korányi Institute of Pulmonology Budapest Hungary; ^2^ Department of Pathology and Experimental Cancer Research Semmelweis University Budapest Hungary; ^3^ Department of Thoracic Surgery, Comprehensive Cancer Center Medical University of Vienna Vienna Austria; ^4^ Karl‐Landsteiner‐Institute for Clinical and Translational Thoracic Surgery Research, Clinic Floridsdorf Vienna Austria; ^5^ Division of Oncology, Department of Internal Medicine and Oncology Semmelweis University Budapest Hungary; ^6^ Department of Thoracic Surgery, University Medicine Essen – Ruhrlandklinik University Duisburg‐Essen Essen Germany; ^7^ Department of Pathology, Forensic and Insurance Medicine Semmelweis University Budapest Hungary; ^8^ Institute of Chemical Technologies and Analytics, Division of Instrumental Analytical Chemistry TU Wien Vienna Austria; ^9^ Center for Cancer Research Medical University of Vienna Vienna Austria; ^10^ Department of Thoracic Surgery National Institute of Oncology‐Semmelweis University Budapest Hungary; ^11^ Department of Translational Medicine Lund University Lund Sweden

**Keywords:** intratumoural drug distribution, laser ablation‐inductively coupled plasma‐mass spectrometry, mass spectrometry imaging, neoadjuvant chemotherapy, platinum, pleural mesothelioma

## Abstract

**Aims:**

Pleural mesothelioma (PM) is a highly aggressive thoracic tumour with poor prognosis. Although reduced tissue drug accumulation is one of the key features of platinum (Pt) resistance, little is known about Pt distribution in human PM.

**Methods:**

We assessed Pt levels of blood samples and surgically resected specimens from 25 PM patients who had received neoadjuvant Pt‐based chemotherapy (CHT). Pt levels and tissue distributions were measured by laser ablation‐inductively coupled plasma‐mass spectrometry and correlated with clinicopathological features.

**Results:**

In surgically resected PM specimens, mean Pt levels of nontumourous (fibrotic) areas were significantly higher (*vs* tumourous regions, *P* = 0.0031). No major heterogeneity of Pt distribution was seen within the tumourous areas. Pt levels correlated neither with the microvessel area nor with apoptosis rate in the tumourous or nontumourous regions. A significant positive correlation was found between serum and both full tissue section and tumourous area mean Pt levels (*r* = 0.532, *P* = 0.006, 95% confidence interval [95% CI] 0.161‐0.771 and *r* = 0.415, *P* = 0.039, 95% CI 0.011‐0.702, respectively). Furthermore, a significant negative correlation was detected between serum Pt concentrations and elapsed time from the last cycle of CHT (*r* = −0.474, *P* = 0.017, 95% CI −0.738‐−0.084). Serum Pt levels correlated negatively with overall survival (OS) (*P* = 0.029).

**Conclusions:**

There are major differences in drug distribution between tumourous and nontumourous areas of PM specimens. Serum Pt levels significantly correlate with full section and tumourous area average Pt levels, elapsed time from the last CHT cycle, and OS. Further studies investigating clinicopathological factors that modulate tissue Pt concentration and distribution are warranted.

## Graphical abstract

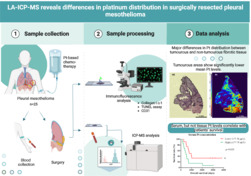

**What is already known about this subject**

- Reduced tissue drug accumulation is one of the key features of chemotherapy resistance.
- Little is known about platinum (Pt) distribution in human pleural mesothelioma (PM).

**What this study adds**

- Pt accumulates in the nontumourous (fibrotic) compartments of PM tissue specimens.
- Serum Pt levels positively correlate with both full tissue section and tumourous area average Pt levels, and negatively correlate with overall survival.
- These findings might lead to the development of optimized treatment protocols in PM.

## 1 INTRODUCTION

Despite efforts regarding early detection and treatment, the overall survival (OS) outcomes of pleural mesothelioma (PM) patients remain poor. The median OS of advanced‐stage patients is usually less than 1 year from diagnosis, and even in early stages the expected 5‐year survival rate is only 15%.^1^ The most widely known negative prognosticators are old age, male sex, poor general condition, advanced disease stage and non‐epithelioid histology.^2^ Importantly, due to its insidious and progressive nature, PM is resectable in only 10‐15% of patients.^3^ In selected cases with favourable prognostic parameters, a multidisciplinary management approach is used.^4^

Until recently, pemetrexed plus platinum (Pt) analogues constituted the main therapeutic approach of PM, associated with a median OS of approximately 12.5 months.^5^, ^6^ In the Mesothelioma Avastin Cisplatin Pemetrexed Study (MAPS), the addition of bevacizumab to the cisplatin‐pemetrexed combination has shown a statistically significant median OS benefit.^7^ As a result, bevacizumab has been incorporated into clinical practice guidelines for PM treatment.^8^ In 2020, immune checkpoint inhibitors (ipilimumab and nivolumab) were approved in the first‐line setting.^9^

However, Pt‐containing drugs still form the backbone of systemic chemotherapy (CHT) in various solid tumours, including PM.^10^ Their major mode of action—the formation of Pt‐DNA adducts—leads to various cellular responses, including replication and cell‐cycle arrest, transcription inhibition, DNA damage response and apoptosis. Pt resistance can be the result of changes in any of these molecular machineries as well as of alterations in chemotherapeutic agent cellular uptake or export.^11^ Furthermore, the phenomenon of limited intratumoural drug penetration also has to be taken into account; abnormal tumour vasculature might result in reduced blood supply, leading to inadequate chemotherapeutic drug delivery.^12^ Attempts to measure intratumoural drug concentrations and efforts to correlate their levels with clinicopathological and vascular parameters are therefore justified.

Studies in several tumour types have emphasized the importance of intratumoural Pt levels. When treating different tumour cell lines with cisplatin, the level of DNA platination significantly correlated with therapy response.^13^ In non‐small cell lung cancer (NSCLC), patients with higher Pt concentration of the homogenized tumour tissue had longer progression‐free survival and OS.^14^ Moreover, in an NSCLC ex vivo explant model, high Pt uptake correlated with improved therapeutic response.^15^

As for PM, Vázquez et al. investigated the in vitro sensitivity of patient‐derived PM cell lines to standard‐of‐care chemotherapeutics and found that they were not intrinsically resistant to cisplatin and gemcitabine.^16^ Interestingly, however, tumours derived from the same cell lines were poorly sensitive to these therapeutic agents in vivo, emphasizing the importance of intratumoural chemotherapeutic drug level measurement in PM. In addition, Giordano et al. showed that when applying a single shot of paclitaxel (PTX), drug distribution differed among cancer models with different histopathological characteristics. In their PM model, fibrotic and necrotic areas showed lower signal intensities than viable tumour regions.^17^ However, to date, little is known about Pt tissue distributions in human PM.

Inductively coupled plasma mass spectrometry (ICP‐MS) is an analytical technique used to identify elements.^18^ Laser ablation devices, which are also commonly used in combination with ICP‐MS instruments (LA‐ICP‐MS), can be used to investigate solid samples too.^19^ Therefore, in our study, we measured the Pt level of blood samples and surgically resected tissue specimens of 25 PM patients who had received neoadjuvant Pt‐based CHT. Moreover, we investigated if these Pt levels correlate with histological and clinicopathological parameters of our PM patient cohort.

## 2 METHODS

### 2.1 Study population and sample collection

Patients with histologically confirmed PM treated with neoadjuvant CHT across Austria and receiving subsequent surgery at the Department of Thoracic Surgery, Medical University of Vienna, Austria between 2011 and 2017 were included in this study. All diagnostic and therapeutic approaches were conducted in accordance with the appropriate National Comprehensive Cancer Network guidelines.^20^, ^21^ Accordingly, based on predefined study aims, all patients received Pt‐based CHT prior to surgery. Of note, CHT administration and subsequent surgery were not necessarily applied in the same institution. The type of surgical approach was chosen according to the international guidelines of the corresponding study period, and tailored to the clinical factors and to the individual surgical judgement and expertise of the surgeon.^20^, ^22^, ^23^ No patients received hypertermic intrathoracic chemotherapy during surgery.^24^ After surgical resection, patients were treated with either CHT, palliative radiotherapy, combined chemoradiotherapy or best supportive care. Clinical data regarding patient age, gender, clinical stage, histological subtype, blood test parameters, treatment and survival were retrospectively collected from medical records and from the Austrian Public Health Insurance Office.

Tissue samples (n = 25) were collected during extrapleural pneumonectomy (EPP) and snap frozen in liquid nitrogen. Blood samples (n = 25) were collected by venous puncture from PM patients prior to surgery into serum separator tubes (Cat No 367985; BD). Centrifugation was performed after a 45‐min clotting time at 1200 × *g* for 10 min at room temperature, aliquoted and then snap frozen in liquid nitrogen. Both blood and tumour samples were stored at −80 °C.

Estimated glomerular filtration rate (eGFR) was calculated based on serum creatinine levels according to Chronic Kidney Disease Epidemiology Collaboration creatinine equation.

### 2.2 Tissue sample preparation and staining

Five consecutive 10 μm sections were cut from frozen tissues at −20 °C. The samples were analysed consecutively with the following methods: (1) terminal deoxynucleotidyl transferase deoxyuridine triphosphate (dUTP) nick end labelling (TUNEL) assay (to examine apoptosis of PM cells); (2) anti‐cluster of differentiation 31 (CD31) staining (to measure microvessel areas [MVAs]); (3) collagen I labelling (to localize collagen‐rich tissues); (4) LA‐ICP‐MS imaging (LA‐ICP‐MSI) (to analyse Pt distribution and Pt levels); (5) haematoxylin and eosin (H&E) staining.

Tissue slides were then digitalized by TissueFAXS (TissueGnostics GmbH) with a 20× objective. The TissueFAX Viewer (TissueGnostics GmbH) software was used to export the scanned images.

The tumour content of our samples was heterogeneous. Thus, the proportion of tumourous and nontumourous tissue areas was measured on H&E‐stained samples using ImageJ software and confirmed by collagen staining. The Pt levels of the whole tissue section and the tumourous and nontumourous regions were also analysed.

MVA and TUNEL signals were evaluated by ImageJ software on the stained (CD31 and TUNEL) tissue sections using entire sections and excluding unspecific signals. The observed parameters were expressed as percentages (area of positive pixels/total area × 100).

Further methods and protocols are described in Supporting Information Data S1. The primary and secondary antibodies used for immunofluorescence labelling are listed in Supporting Information Table S1.

### 2.3 Tissue and serum Pt measurements

A quadrupole ICP‐MS instrument (Thermo iCAP Qc, Thermo Fisher Scientific) was used for the measurements of different elements. For solid sampling experiments, a commercially available laser ablation system with a frequency quintupled 213 nm Nd:YAG laser was employed (New Wave 213, ESI). To avoid higher risk of signal distortions, the used washout cell was held above the actual ablation site to guarantee fast washout times (below 1 s). As a carrier gas, helium was used to wash out the cell. Next, helium and the make‐up gas, argon, were mixed on introduction to the plasma. ^13^C, ^31^P, ^34^S, ^194^Pt, ^195^Pt and ^197^Au were detected with ICP‐MSI. The calibration of the instrument and the optimization of the method, as well as the procedure applied for signal correction, were described previously.^19^, ^25^, ^26^ In brief, a thin gold layer was sputtered onto the sample surface, as a pseudo‐internal standard, and the measured Pt‐signals were corrected using the observed ^197^Au signals.

For data acquisition, Qtegra software (Thermo Fisher Scientific) was used. After MS data export, serum sample analysis was performed using Microsoft Excel software. The raw data of solid samples were transformed into two‐dimensional images and analysed by ImageLab 2.18 (Epina GmbH) software.

Further ICP‐MS settings for serum and tissue analyses are described in Supporting Information Data S1. The typical measurement settings of solid samples are summarized in Supporting Information Table S2.

### 2.4 Statistical analysis

To compare serum and tissue ^195^Pt signals with clinicopathological data, statistical analysis was performed with GraphPad Prism 5 (GraphPad Software, Inc.), Microsoft Excel and R version 4.0.4 (R Foundation for Statistical Computing). Data distribution was verified by the Kolmogorov‐Smirnov normality test. Tissue and serum Pt levels were analysed with regard to clinicopathological variables by the Wilcoxon signed rank test, the Mann‐Whitney *U* test, Student's *t*‐test or the Kruskal‐Wallis test (and Dunn's test). The association between serum and tissue Pt levels and other continuous variables was evaluated using the Spearman rank correlation. Statistical analysis of survival was done by the log‐rank (Mantel‐Cox) test. Differences were considered statistically significant at *P* < .05.

### 2.5 Ethics statement

This study was conducted in accordance with the guidelines of the Helsinki Declaration (as revised in 2013) of the World Medical Association and the Good Scientific Practice guidelines of the Medical University of Vienna with the approval of the national level ethics committee (Medical University of Vienna, EK#: 904/2009). All patients provided written informed consent and were listed anonymously in securely handled databases.

## 3 RESULTS

### 3.1 Clinicopathological characteristics and Pt levels

The full cohort comprised 21 (84%) epithelioid and four (16%) nonepithelioid (ie, biphasic or sarcomatoid) PMs. The median age of all cases was 64.5 years (range 33‐78 years) and patients were predominantly male (76%). At the time of sampling, 16 (64%) and nine (36%) patients had International Mesothelioma Interest Group (IMIG)/TNM stage I‐II and stage III‐IV disease, respectively. Data are shown in Supporting Information Table S1.

**FIGURE 1:**
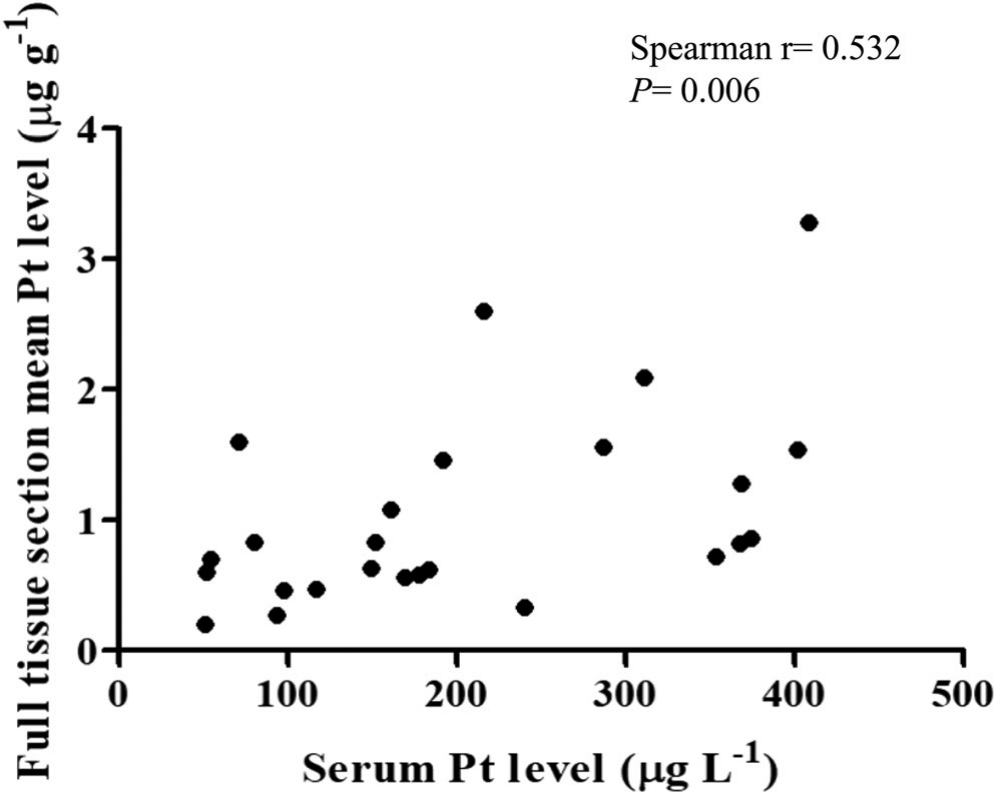
Scatter plot showing statistically significant positive correlation between serum and full tissue section mean Pt levels (Spearman *r* = 0.532, *P* = .006). Pt, platinum.

Serum Pt concentrations ranged between 50.7 and 408.4 μg L^−1^, with a mean value of 205.1 μg L^−1^. The mean full tissue section Pt level was 1.04 μg g^−1^ (range 0.2‐3.28 μg g^−1^). A statistically significant positive correlation was found between serum and full tissue section average Pt levels (Spearman *r* = 0.532, *P* = .006; Figure 1).

### 3.2 Pt accumulates in the fibrotic, nontumourous regions of surgical PM tissue specimens

The histological composition of surgically resected PM specimens was heterogeneous. H&E and collagen staining revealed, that while some samples only contained tumourous areas (n = 7), others were mixed in terms of histological composition, with variable amounts of collagen rich fibrotic tissue mixed with viable tumour compartments (n = 18).

**FIGURE 2:**
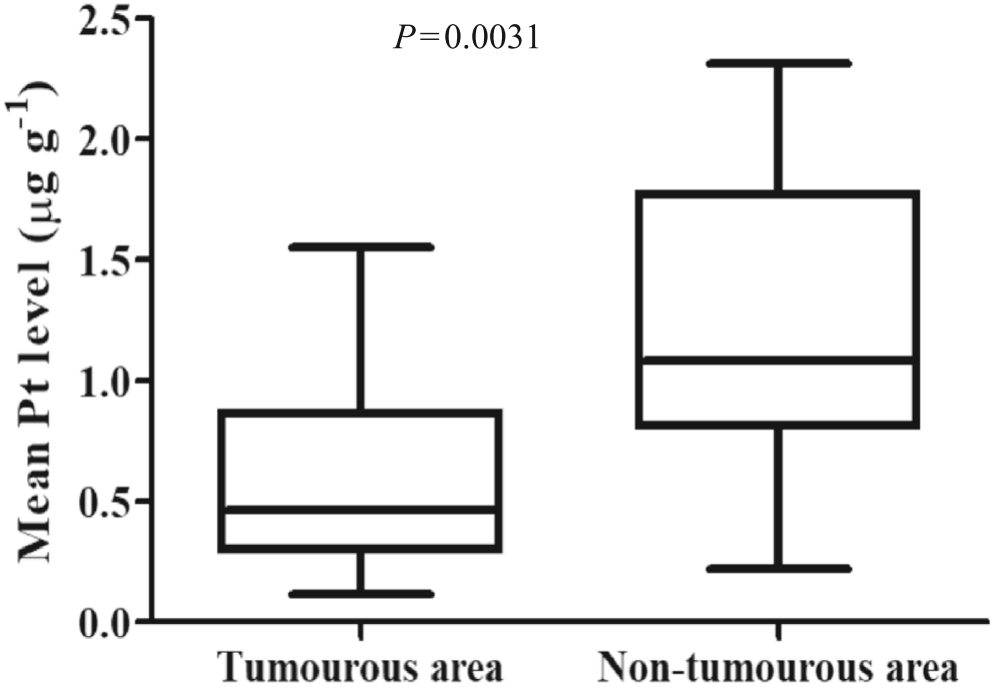
Pt levels of tumourous and nontumourous tissue compartments. The mean Pt level was significantly higher in nontumourous areas as compared to tumourous regions (1.23 μg g^−1^ *vs* 0.83 μg g^−1^, respectively; Mann‐Whitney *U* test, *P* = .0031).

When analysing the Pt distribution in tumourous *vs* nontumourous regions, we found that Pt accumulated in the fibrotic (ie, nontumourous) areas. Accordingly, the mean Pt level was significantly higher in nontumourous areas compared to tumourous regions (1.23 μg g^−1^ *vs* 0.83 μg g^−1^, respectively, Mann‐Whitney *U* test, *P* = .0031; Figure 2).

**FIGURE 3:**
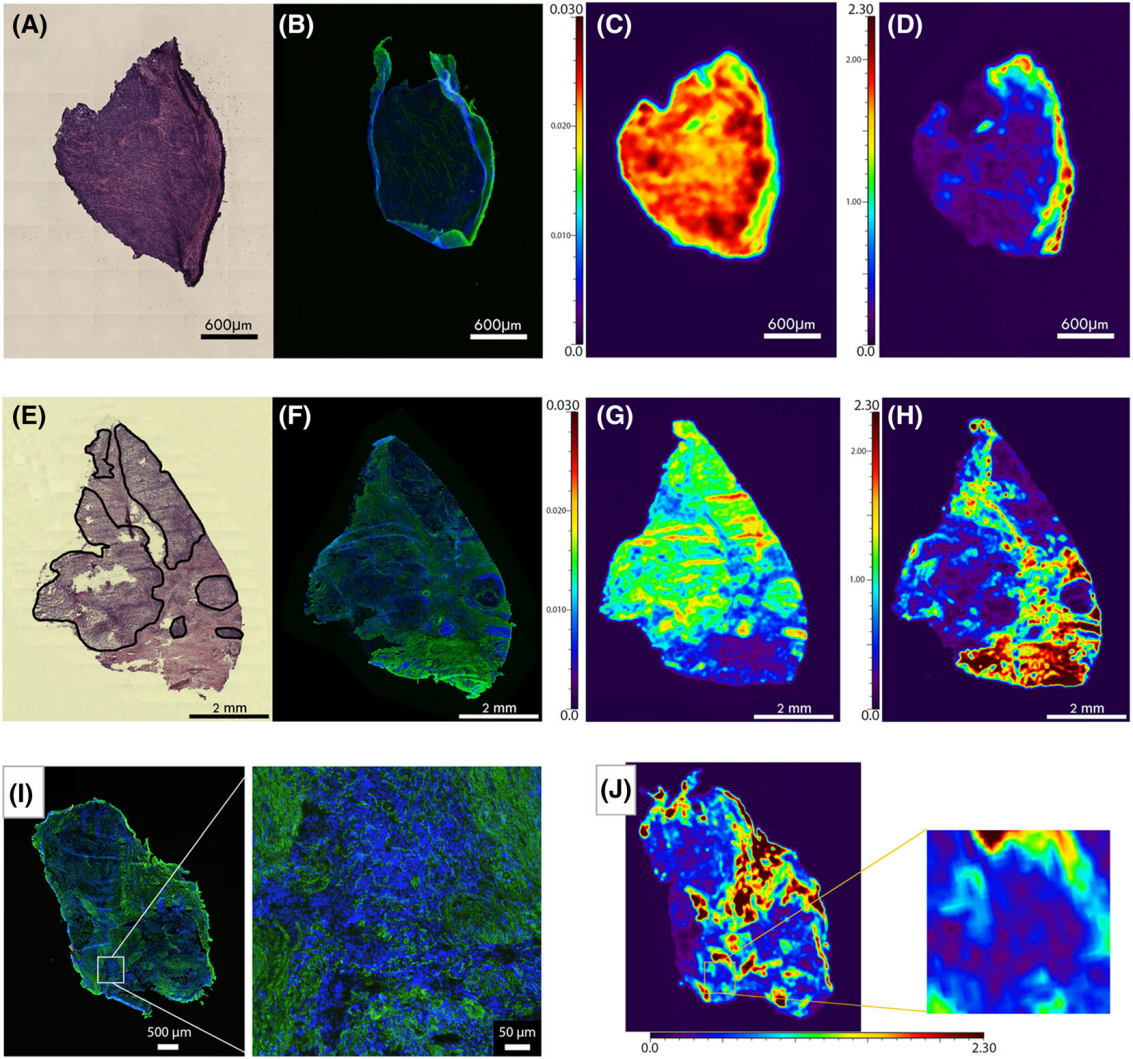
Representative images of collagen, P and Pt distribution in tumourous and nontumourous compartments of PM specimens. H&E (A) and collagen (B) stainings of the tumourous area reveal low collagen content. Meanwhile, the same area exhibits high P (C) and low Pt (D) signals as measured by LA‐ICP‐MSI. Comparative analysis of tumourous and nontumourous (ie, fibrotic) areas of another PM sample (E‐H) show considerable differences in collagen distribution according to H&E (E) and collagen (F) staining. Tumourous compartments are delineated with solid black lines (E). LA‐ICP‐MSI revealed that tumourous areas contain P (G) and Pt (H) to a greater and lesser extent, respectively (*vs* the collagen‐rich nontumourous compartments). Similar tendencies are seen in a third PM specimen (I, J), as Pt accumulates in the nontumourous, collagen‐rich fibrotic area of the tissue sample. Blue, nuclear staining with DAPI; green, collagen staining with anti‐collagen type I α‐1. The colour bar scale represents the P and Pt signal intensities of a given compartment as measured by LA‐ICP‐MSI. Cold colours, low signal intensities; warm colours, high signal intensities. H&E, haematoxylin and eosin; LA‐ICP‐MSI, laser ablation‐inductively coupled plasma‐mass spectrometry imaging; P, phosphorous; PM, pleural mesothelioma; Pt, platinum.

Moreover, as shown in Supporting Information Figure S1, when analysing the paired tumourous and nontumourous compartments within the same tissue specimen, we also found a statistically significant difference in mean Pt levels (Wilcoxon matched‐pairs signed‐rank test, *P* = .0003). Representative images demonstrating tissue heterogeneity by H&E staining, collagen, P and Pt distribution are shown in Figure 3.

### 3.3 Tumour tissue Pt levels do not correlate with angiogenesis and apoptosis in human PM

Preclinical studies suggest that apoptosis defects and increased tumour vascularization are characteristic features of PM progression and chemoresistance.^27^, ^28^ In our cohort, the mean tumourous and nontumourous MVAs were 3.66% and 3.78%, respectively, and they did not differ statistically (Spearman *r* = 0.083, *P* = .751). As shown in Supporting Information Table S2, no significant correlation was found between tumour tissue Pt levels and MVA either in the tumourous (Spearman *r* = −0.122, *P* = .56) or in the nontumourous (Spearman *r* = −0.222, *P* = .392) regions.

To determine the rate of apoptotic cells, we performed a TUNEL assay. However, we did not detect any correlations between Pt levels and apoptotic rate in tissue samples (tumour areas: Spearman *r* = −0.144, *P* = .501; nontumourous areas: Spearman *r* = −0.163, *P* = .546).

### 3.4 Association between serum and tissue Pt levels and clinicopathological parameters

**FIGURE 4:**
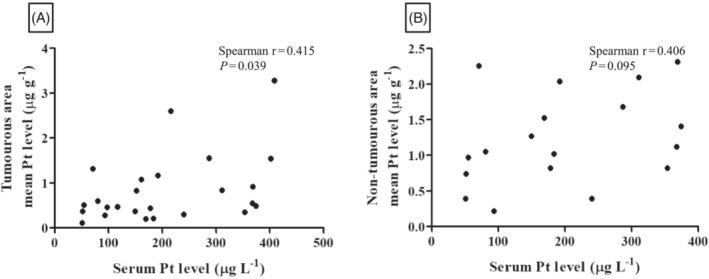
Correlation patterns between the Pt level of serum and different tissue compartments. (A) Scatter plot showing a statistically significant positive correlation between serum and tumoural Pt levels (Spearman *r* = 0.415, *P* = .039). (B) No statistically significant correlation was found between serum Pt concentrations and the Pt levels of the nontumourous area (Spearman *r* = 0.406, *P* = .095). Pt, platinum.

Serum Pt concentration correlated with Pt levels in tumourous areas to a greater degree than in nontumourous regions (Spearman *r* = 0.415, *P* = .039 *vs r* = 0.406, *P* = .095, respectively). Importantly, only the correlation between serum and tumourous area Pt levels remained statistically significant in these subgroup analyses (Figure 4).

No significant differences were found in Pt levels with regards to age, gender, histological subtype and tumour pathological stage in either tissue samples or serum (Supporting Information Table S1).

Both serum and tumour tissue Pt levels might depend on the time passed between the last cycle of CHT and sampling (ie, surgery). As expected, a statistically significant negative correlation was found between serum Pt levels and elapsed time from the last CHT (Spearman *r* = −0.474, *P* = .017). In contrast, no such correlation was found between the elapsed time and the full tissue section mean Pt levels (Spearman *r* = −0.24, *P* = .249). Similarly, the elapsed time from the last CHT did not correlate with either the tumourous (Spearman *r* = −0.261, *P* = .208) or the nontumourous tissue Pt levels (Spearman *r* = −0.12, *P* = .636).

Next, to examine whether kidney function influences Pt levels, calculated eGFR (both pre‐CHT and post‐CHT/pre‐surgery) values were compared to serum Pt concentrations, and full‐, tumourous‐ and nontumourous PM tissue Pt levels, but no correlations were found (data not shown). Interestingly, a trend towards a mild negative correlation was observed between pre‐operational eGFR levels and nontumourous Pt levels (Spearman *r* = −0.457, *P* = .065).

With regard to the number of CHT cycles, the vast majority of patients (n = 19) received three cycles of neoadjuvant CHT, thus the case numbers in the other groups were too low to adequately address the question of whether the number of CHT cycles influences serum or tissue Pt levels.

As for the type of chemotherapeutic agents, most patients included in our study received cisplatin (n = 16), whereas others were treated with carboplatin (n = 7). We found that PM patients treated with carboplatin had comparable serum (Mann‐Whitney, *P* = .3) and tissue Pt (Mann‐Whitney, *P* = .867, 0.442, 0.571 for full‐, tumourous‐ and nontumourous tissue compartments, respectively) levels to those receiving cisplatin (note that two patients received both; data shown in Supporting Information Table S3).

### 3.5 Serum but not tumour tissue Pt levels correlate with survival of PM patients

**FIGURE 5:**
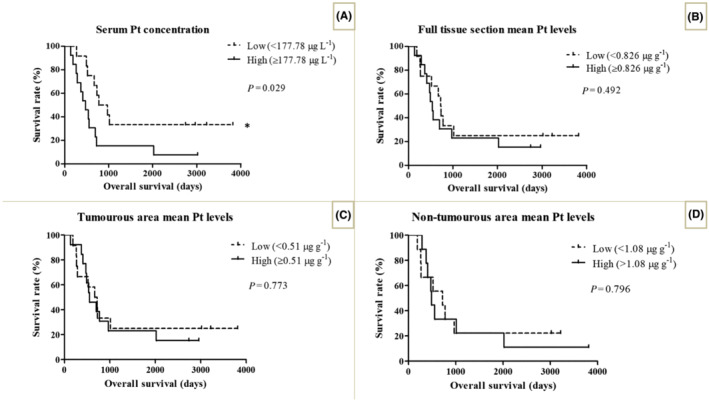
Kaplan‐Meier estimates for OS according to serum and tissue mean Pt levels. (A) Patients with high serum Pt levels (≥177.78 μg L^−1^) exhibited significantly worse median OS than those with low serum Pt levels (<177.78 μg L^−1^) (log rank test, *P* = .029). The Pt level of the full tissue section, the tumourous area and the nontumourous compartment did not have a significant impact on OS (log rank test, *P* values were .492, .773 and .796, respectively). OS, overall survival; Pt, platinum. *P < .05.

Next, we examined if serum or tissue Pt levels have prognostic significance in PM. Using the median serum concentration (177.78 μg L^−1^) as a cut‐off, we found that patients with low serum Pt concentration had significantly longer OS than those with high serum Pt concentration (median OS was 873 days *vs* 476 days, respectively, *P* = .029; Figure 5A). Nevertheless, as shown in Figure 5B–D, no significant differences were found in OS with regard to the tissue Pt levels of full‐, tumourous‐ and nontumourous‐areas (log‐rank test, median cut‐offs: 0.826, 0.51, 1.08 μg g^−1^; *P* = .492, *P* = .773 and *P* = .796, respectively).

Lastly, we compared the apoptotic rate and MVA of tumourous areas with OS, but no significant correlations were revealed (Spearman *r* = −0.354, *P* = .09 and *r* = 0.298, *P* = .157, respectively; Supporting Information Figure S2).

## 4 DISCUSSION

PM is a rare but devastating disease that is largely caused by asbestos exposure.^29^ Pt compounds have been used for cancer treatment since the 1970s and even nowadays, in the era of targeted‐ and immunotherapies, they form the backbone of systemic treatment in several solid tumours, including PM.^10^ Nevertheless, their contribution to OS improvement remains moderate.^30^ Importantly, a pivotal cause of therapeutic resistance may be insufficient drug penetration into the tumour tissue itself.^12^ In the current study, we assessed the Pt levels of blood and surgically resected tissue samples of PM patients treated with neoadjuvant Pt‐based CHT and correlated the measured Pt levels with histological and clinicopathological parameters.

An adequate blood supply is regarded as essential for the growth of solid tumours.^31^ To ensure the necessary amount of oxygen and nutrients, the tumour vascular network can be formed by various mechanisms.^32^ Indeed, increased vascularization has been regarded as a negative prognosticator in several tumour types,^33^ including PM.^27^, ^34^ On the other hand, optimal drug delivery also relies strongly on tumour vascularization.^12^, ^35^ The beneficial effects of radio‐, chemo‐ and immunotherapy also depend on the intratumoural circulation.^36^, ^37^ Accordingly, we investigated the association between tissue MVA and Pt penetration into the tumour, as well as patient survival. Importantly, MVA influenced neither of these parameters in our study. It is important to mention, however, that increased intratumoural vascularity does not necessary improve oxygenation, drug delivery and therapeutic response, as tumoural blood vessels are structurally and functionally abnormal.^38^ This may contribute to a hostile microenvironment and to the selection of more aggressive tumour cells.^38^ This led Rakesh Jain to the elaboration of the “vessel normalization theory”, whereby treatment with an anti‐VEGF antibody (bevacizumab) normalizes the chaotic tumour blood vessel network, leading to increased drug delivery.^39^ Based on this hypothesis, anti‐angiogenic agents became widely studied and introduced to clinical practice in PM and several other malignancies. However, lately serious concern has been raised that bevacizumab can reduce the uptake of chemotherapeutic drugs.^40^ Specifically, Cesca et al. found lower intratumoural PTX concentrations in ovarian and colon carcinoma models when pretreated with bevacizumab.^41^ As expected, drug penetration was inadequate in poorly vascularized or necrotic parts of tumours receiving only PTX. However, bevacizumab pretreatment was accompanied by a decrease in intratumoural necrosis and consequently with a more homogenous PTX distribution. Moreover, PTX was cleared more slowly in bevacizumab‐pretreated tumours. Interestingly, lower intratumoural PTX concentrations in the bevacizumab‐pretreated group still resulted in a synergistic therapeutic effect, suggesting that the slower clearance contributed to prolonged and efficient tumour drug exposure.

In our study, no significant difference was detected between the MVA of tumourous and nontumourous tissue areas considering the whole patient population. However, it is important to mention that only two patients received bevacizumab as part of their neoadjuvant treatment in our cohort. Although no outstanding values of MVA or tissue Pt levels were detected in bevacizumab‐treated patients, given their low number it cannot be estimated if bevacizumab treatment influenced tissue Pt levels in our study. Of note, vascular tortuosity and permeability, which may also influence drug delivery, were not assessed in the current study.

Even if drug delivery to the tumour tissue itself is sufficient, several processes can lead to inadequate therapeutic response. We therefore investigated whether tissue Pt levels correlate with response to CHT. We found that tissue Pt levels (full, tumourous and nontumourous areas) did not show correlation with either the apoptotic rate of tumour cells (as determined by TUNEL labelling) or the patients' OS. These findings are in contrast to the literature, as high intratumoural Pt level was identified as a favourable prognostic factor in different tumour types.^14^, ^15^, ^42^, ^43^ Moreover, in a recent study on cisplatin‐treated NSCLC ex vivo explant cultures, Ki67 labelling (cell proliferation) and poly‐ADP‐ribose polymerase (PARP) expression (cell death) also correlated with intratumoural Pt levels.^15^ Our results indicate, that in addition to inadequate penetration into the tumour tissue,^13^ other types of resistance mechanisms may also play a key role in the limited effectiveness of Pt agents in PM patients.^11^, ^44^

Although spatial drug distribution can affect therapeutic response, most studies investigated the level of chemotherapeutic agents only in cells or tissue homogenates.^45^ As LA‐ICP‐MSI is suitable for the visualization of Pt spatial distribution, we investigated whether there is a pattern in Pt tissue distribution that might affect the therapeutic response in human PM. We found that Pt spatial distribution is heterogeneous in the tissue samples and nonmalignant, collagen‐rich fibrotic areas show higher Pt levels than tumourous compartments. Similar findings were reported by Chang et al. in a patient‐derived xenograft model of pancreatic cancer. In their study, Pt accumulated in the collagen fibres of both the tumour stroma and nonmalignant tissues (skin, small intestine and kidney) of cisplatin‐treated tumour‐bearing mice. In contrast, Pt levels remained relatively low in pancreatic cancer cells.^46^ These authors suggested that the collagen‐bound Pt may be slowly released, and as a result the adjacent tumour cells are constantly exposed to metronomic CHT, leading to a therapeutic response. Similarly, Cao et al. demonstrated that Pt binds to the collagen compartment of gastric cancer tissue samples. In their cohort, both collagen content and binding of Pt to collagen fibres were significantly higher in those responding to therapy compared to the therapy‐resistant cases.^42^ Pt compounds interact with proteins containing free methionine and cysteine side chains through the thiol groups of the amino acids.^47^ Moreover, Pt also binds to the nitrogen of imidazole on the histidine residue of proteins.^48^ Given that histidine and methionine also present in collagen fibres, it is likely that the Pt‐collagen bond is realized through these amino acids.^49^ On the other hand, it is well established that cancer‐associated fibroblasts can also decrease intratumoural Pt concentrations by altering the tumour microenvironment or releasing cysteine and glutathione, which bind Pt through their thiol groups.^50^, ^51^ Given that stromal collagen is produced by cancer‐associated fibroblasts,^52^, ^53^ co‐localization of cysteine and glutathione with collagen could also contribute to the observed accumulation of Pt in the fibrotic collagen‐rich tumour stroma. The possibility of this phenomenon needs to be further investigated.

Serum Pt concentrations can fundamentally influence tissue Pt levels and may explain the differences in patient survival, therefore we correlated serum Pt levels with that of the tissues and, moreover, with the patients' OS and found that low serum Pt concentration was associated with lower full and tumour tissue Pt levels and, interestingly, with better OS. It is well known that Pt can accumulate in nonmalignant tissues, resulting in long‐term treatment‐related adverse events associated with CHT.^54^, ^55^ Notably, biologically active cisplatin can be detected in the peripheral blood of patients even years after CHT administration.^56^, ^57^ The collagen of healthy tissues was speculated as a potential source.^46^ Based on these findings, a possible explanation of our results might be that in patients with low serum Pt levels the drug accumulated in the collagen‐rich nonmalignant tissues. This might subsequently lead to a constant low‐dose metronomic Pt exposure even after the removal of the tumour tissue, thus hindering tumour recurrence. However, this hypothesis clearly requires further investigation.

Since a wide range of additional factors can influence the adsorption, distribution, metabolism and excretion of therapeutic agents, we examined whether the clinicopathological characteristics of our patients affected serum and tissue Pt levels. However, we found no correlation between tissue or serum Pt levels and age, gender, histological subtype, tumour pathological stage, renal clearance or the type of Pt agent. These findings are consistent with the literature, as none of these parameters had an impact on tissue Pt levels of NSCLC or gastric cancer patients.^14^, ^43^ Moreover, although cisplatin and carboplatin levels have not been directly compared in PM tissues yet, as they show equivalent survival rates, it is presumable that they have similar biological activities in PM.^58^

As expected, serum Pt concentrations negatively correlated with the elapsed time between the last CHT cycle and sample collection. Interestingly, however, no such correlation could be detected in the case of tissue Pt levels, suggesting that tissue‐bound Pt is slowly released. Indeed, Tothill et al demonstrated that nontumourous tissue Pt levels remain comparable 1‐17 months after the last CHT dosage.^55^ Given that surgery was performed within 11 weeks (median 4 weeks) after the completion of neoadjuvant CHT, our timescale might have been too short to significantly impact tissue Pt levels.

Although the experimental approaches used are not yet part of routine clinical practice, the current study has the potential to provide a unique framework for personalized clinical pharmacological approaches in the future. Specifically, by providing insights into intratumoural drug penetration and distribution, our results might contribute to CHT dosage optimization. Moreover, since serum Pt concentrations correlate with tissue Pt levels, intratumoural drug penetration can be estimated even using noninvasive approaches. After further validation, may allow us in the future to study the dynamic changes in drug penetration and estimate therapeutic efficacy.

The present study has certain limitations that need to be addressed in future settings. First, although we collected a unique cohort of matched blood and surgically resected samples, the number of included patients remained relatively small. Nonetheless, given that PM is a relatively rare disease and only a fraction of patients are eligible for multimodality treatment, including neoadjuvant Pt‐based CHT, this can be regarded as a rather large cohort. Second, the heterogeneity of the study population's clinicopathological characteristics made the subgroup analyses difficult to accomplish. Lastly, only a fraction of patients received their neoadjuvant CHT in our department, thus causing a potential delay in surgical resection. Consequently, patients were subjected to surgery within a relatively large timescale from the last cycle of CHT. This might have influenced Pt levels, but given the heterogeneity of our cohort, multivariate statistical analyses were not feasible. Given these study limitations, we believe that our findings in their current form are hypothesis‐generating and further validation studies are needed.

To the best of our knowledge, this is the first study investigating serum and tissue Pt levels and tissue distributions of surgically treated PM patients using LA‐ICP‐MS. Our results revealed major differences in Pt distribution between tumourous and nontumourous fibrotic areas of surgically resected PM specimens, with nontumourous fibrotic areas showing significantly higher mean Pt levels. We also demonstrated that serum Pt levels significantly correlate with full tissue specimen and tumourous area Pt levels, with elapsed time from the last CHT cycle and with OS. These findings might represent a step forward in our understanding of intratumoural drug penetration and distribution, and might therefore lead to the development of optimized treatment protocols in PM.

## AUTHOR CONTRIBUTIONS

**Anna Tisza**: Investigation; formal analysis; data curation; visualization; writing—original draft; writing—review and editing. **Thomas Klikovits**: Investigation; resources; formal analysis; data curation. **Michal Benej**: Investigation. **Szilvia Torok**: Visualization; writing—original draft; writing—review and editing. **Beata Szeitz**: Formal analysis; writing—review and editing. **Zsuzsanna Valko**: Investigation. **Zsolt Megyesfalvi**: Writing—review and editing. **Mir Alireza Hoda**: Methodology; resources. **Balazs Hegedus**: Conceptualization; writing—review and editing. **Maximilian Bonta**: Methodology; investigation; validation. **Winfried Nischkauer**: Investigation; validation. **Konrad Hoetzenecker**: Methodology; resources. **Andreas Limbeck**: Methodology; resources; writing—review and editing; funding acquisition. **Karin Schelch**: Writing—review and editing. **Viktoria Laszlo**: Conceptualization; methodology; validation; visualization; data curation; writing—original draft; project administration. **Balazs Dome**: Conceptualization; resources; supervision; writing—review and editing; funding acquisition.

## CONFLICT OF INTEREST STATEMENT

The authors declare no conflict of interest.

## Supporting information


**Supporting Information Figure S1** Paired analysis of tumourous *vs* nontumourous compartments of the same tissue specimen reveal significant differences in mean Pt levels (Wilcoxon matched‐pairs signed‐rank test, *P* = 0.0003). Pt, platinum

**Supporting Information Figure S2** Scatter plots for OS according to the (A) apoptotic area and (B) MVA in the tumourous compartments. Statistically significant correlation was not found between OS and apoptotic area (Spearman *r* = −0.354, *P* = 0.09) or between OS and MVA (Spearman *r* = 0.298 *P* = 0.157). OS, overall survival; MVA, microvessel area

**Supporting information data S1** Supplementary materials

**Supporting information Table S1** Pt levels of full tissue specimens and serum according to patients' characteristics
**Supporting information Table S2** Vascularization and mean Pt levels of tumourous and nontumourous tissue areas (Mann‐Whitney test)
**Supporting information Table S3** Pt levels with regards to the type of administered chemotherapeutic agent: cisplatin *vs* carboplatin (Mann‐Whitney test)

## Data Availability

The data that support the findings of this study are available from the corresponding author upon reasonable request. Some data may not be made available because of privacy or ethical restrictions.
