# Supplementary figures and images for: Laser ablation‐inductively coupled plasma‐mass spectrometry analysis reveals differences in chemotherapeutic drug distribution in surgically resected pleural mesothelioma
Source: Br J Clin Pharmacol. 2023 Jul 14;89(11):3364–74. doi: 10.1111/bcp.15813 (PMC10952999; doi:10.1111/bcp.15813)

$P=0.0003$

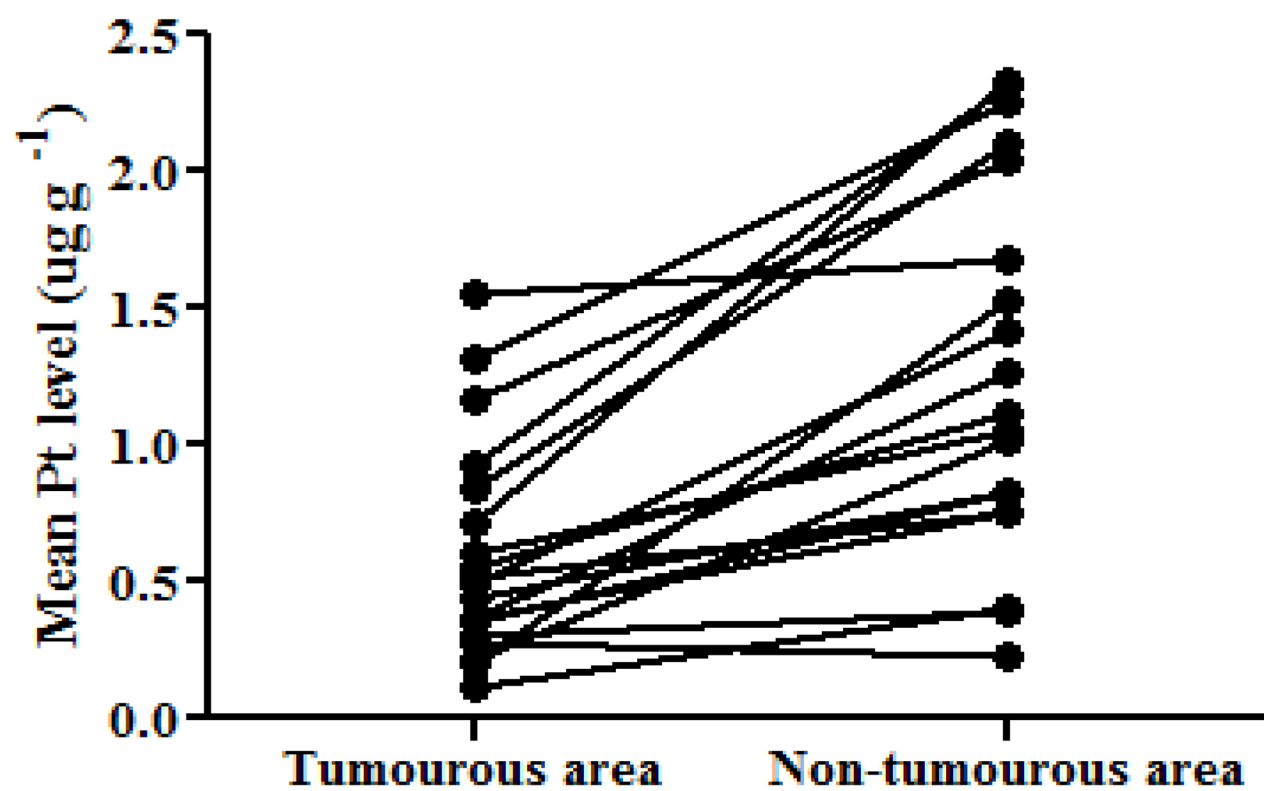

Supplement: Supplementary file 1 — Supporting Information Figure S1 Paired analysis of tumourous vs nontumourous compartments of the same tissue specimen reveal significant differences in mean Pt levels (Wilcoxon matched‐pairs signed‐rank test, P = 0.0003). Pt, platinum [file BCP-89-3364-s002.pdf]

A

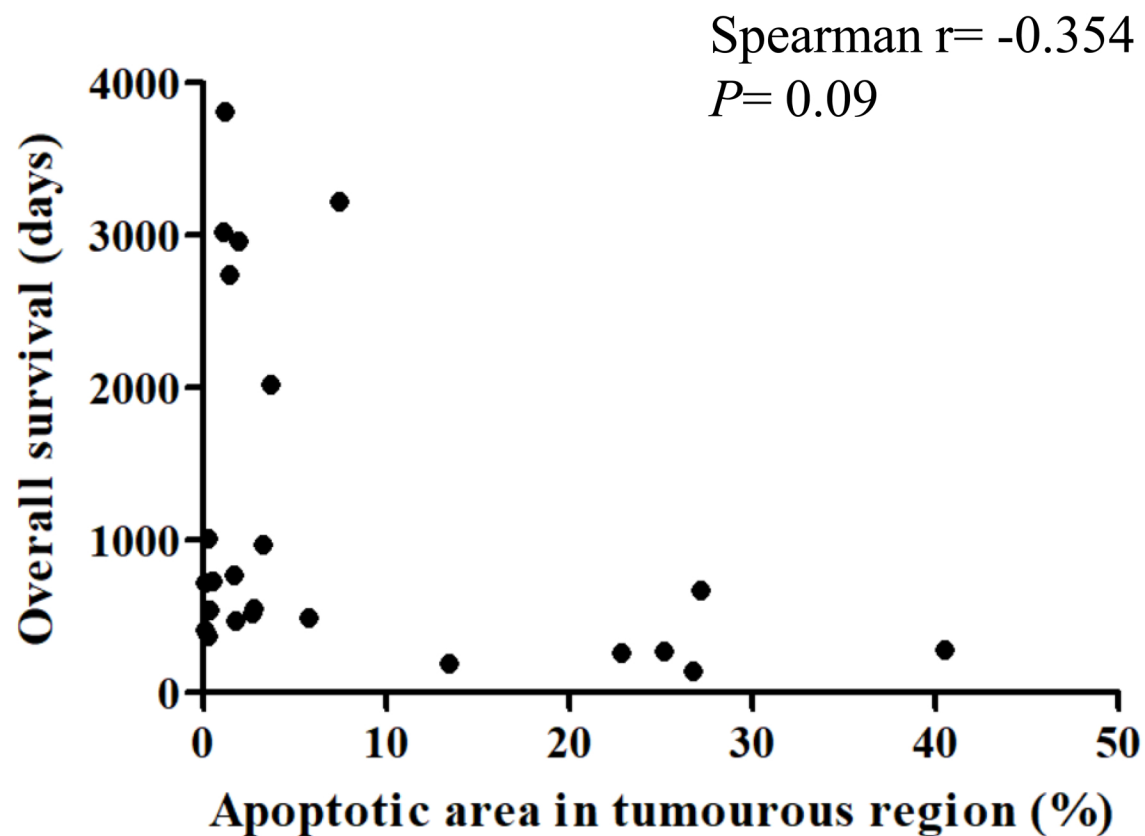

B

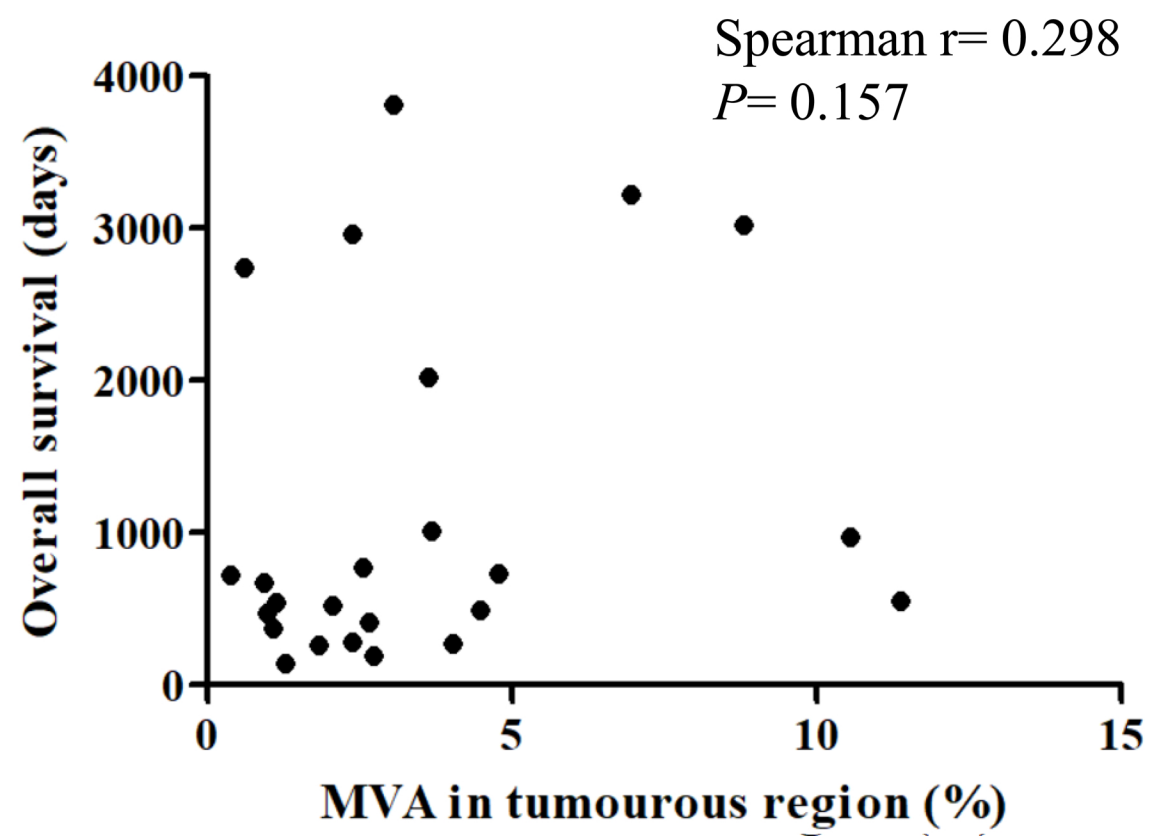

Supplement: Supplementary file 2 — Supporting Information Figure S2 Scatter plots for OS according to the (A) apoptotic area and (B) MVA in the tumourous compartments. Statistically significant correlation was not found between OS and apoptotic area (Spearman r = −0.354, P = 0.09) or between OS and MVA (Spearman r = 0.298 P = 0.157). OS, overall survival; MVA, microvessel area [file BCP-89-3364-s004.pdf]
