# Supplementary material for: Laser ablation‐inductively coupled plasma‐mass spectrometry analysis reveals differences in chemotherapeutic drug distribution in surgically resected pleural mesothelioma
Source: Br J Clin Pharmacol. 2023 Jul 14;89(11):3364–74. doi: 10.1111/bcp.15813 (PMC10952999; doi:10.1111/bcp.15813)
Supplement: Supplementary file 3 — Supporting information data S1 Supplementary materials [file BCP-89-3364-s001.docx]

**Supplementary Materials**

1. **Tissue sample preparation**
   1. **Cryosectioning**

Frozen tissues were attached to a sample holder with Shandon^TM^ Cryomatrix^TM^ (CAT: 6769006; Thermo Fisher Scientific, Waltham, MA, USA) and 5 consecutive 10 µm sections were prepared using a Leica CM3050 S Research Cryostat (Leica CM3050 S, Leica Biosystems, Danaher Corporation, Wetzlar, Germany) at -20 °C. The sections were collected on Superfrost ULTRA Plus glass slides (CAT: J3800AMNZ; Thermo Fisher Scientific, Waltham, MA, USA) and further treated as described below.

- 1. **Immunofluorescent labeling**

Tissues were fixed at -20 °C in methanol and washed with phosphate buffer saline (PBS). Then, samples were blocked with 2% milk powder (Blotting Grade Blocker Non-Fat Dry Milk; CAT: 170-6404; BioRad; Hercules, California, USA) dissolved in PBS. Thereafter, primary antibodies (anti-CD31 or anti-Collagen I alpha-1) were diluted in PBS mixed with 1% milk powder and tissue specimens were incubated with the primary antibodies for 1 h at room temperature (RT). Sections were then washed with PBS, and labeled with appropriate fluorescent secondary antibodies for 30 min at RT. PBS washing of the secondary antibody was followed by the application of DAPI (4',6-diamidino-2-phenylindole) solution (1:1000, EN62248; Thermo Fisher Scientific, Waltham, MA, USA, 10 min, RT) to visualize nuclei. After further washing steps, the sections were covered with Fluoromount-G® (0100-01, SouthernBiotech; Birmingham, USA). Specifications of the used antibodies and nucleic acid stain are shown in Supplementary Materials Table 1.

**Supplementary Materials Table 1. Specifications of different antibodies and nucleic acid stain.**

| **Antibody** | **Host, specificity** | **Company** | **Catalog number** | **RRID** | **Dilution** |
| --- | --- | --- | --- | --- | --- |
| **Primary** | | | | | |
| anti-CD31 | Mouse,  Anti-Human monoclonal | DAKO, Santa Clara, CA USA | M0823 | - | 1:50 |
| anti-Collagen I alpha-1 | Rabbit,  Anti-Human polyclonal | Novus, Abingdon, UK | NB600-408 | AB_10000511 | 1:500 |
| **Secondary** | | | | | |
| Rhodamine (TRITC) | Donkey,  Anti-Mouse polyclonal | Jackson ImmunoResearch Laboratories, INC; West Grove, PA, USA | 715-025-150 | AB_2340766 | 1:500 |
| Fluorescein (FITC) | Donkey  Anti-Rabbit polyclonal | Jackson ImmunoResearch Laboratories, INC; West Grove, PA, USA | 711-095-152 | AB_2315776 | 1:500 |
| **DNA-staining** | | | | | |
| DAPI | - | Thermo Fisher Scientific, Waltham, MA, USA | EN62248 | - | 1:10000 |

- 1. **TUNEL assay**

For the TUNEL (TdT-mediated dUTP-biotin nick end) assay, samples were first fixed in 4.5% paraformaldehyde (Roti TM Histofix 4.5%, CAT: 48625143; Carl Roth GmbH; Karlsruhe, Germany), for 20 min at RT, followed by washing for 30 min in PBS. Afterwards 0.1% TritonX-100 (CAT: T8787-100ML; Sigma-Aldrich, St. Louis, MO, USA) and 0.1% Na-citrate were mixed in PBS on ice and were applied to the surface of the sections for 2 min. Samples were then washed three times for 5 min in PBS. TUNEL reaction mixture was prepared from TUNEL Kit (In Situ Cell Death Detection Kit; CAT: 11684795910; Roche, Basel, Switzerland) according to the manufacturers’ recommendation and was applied for 1 h at 37 °C in dark. Samples were then washed three times for 5 min in PBS, and DAPI staining was applied for 10 min. After the last washing step in PBS slides were covered with Fluoromount-G® (CAT: 0100-20, SouthernBiotech, Birmingham, AL, USA).

- 1. **Hematoxylin & eosin staining**

For haematoxylin and eosin (H&E) staining, sections were rinsed in distilled water (5 min) followed by staining with Harris’ hematoxylin (CAT: HX71788774; Merck, Darmstadt, Germany) for 1 min. Thereafter, samples were rinsed in Scott’s solution (CAT: 11192.01000; Morphisto, Frankfurt, Germany) for 10 min and counterstained with Eosin (CAT: CS701; Dako, Glostrup, Denmark) for 30 sec. This was followed by a dehydrating process: rinsing the sections first in 96%, then in 100% ethanol (CAT: 20821.330; VWR; Radnor, Pennsylvania, USA), both twice for 3 min. Next, sections were rinsed in xylene (CAT: X/0250/15; Thermo Fisher Scientific, Waltham, MA, USA) for 2x5 min, and mounted with Entellan (CAT: HX72093761; Merck, Darmstadt, Germany).

1. **ICP-MS measurements**
   1. **Blood sample preparation**

0.15 mL of the serum samples were transferred into a 10-mL polypropylene vial. Next, 2 mL concentrated HNO_3_ and 0.5 mL H_2_O_2_ (30%) were added to serum samples for 6 h at 80°C. After cooling down, water (18.2 MΩ cm, Easypure II system, Thermo Fisher Scientific, Waltham, MA, USA) was added to the samples to obtain a final volume of 10 mL. During this dilution step, Indium was added to the mixture at a final concentration of 1 ng mL^-1^ as internal standard.

- 1. **Calibration of serum samples**

A matrix-matched calibration was performed to account for possible matrix-effects, which could be caused by remaining ‘organic’ sample constituents or by the highly acidic pH. 4 mL digested sample was taken from each vial, and thoroughly mixed. This “pooled sample”, which contains the matrix-constituents of all samples, acts as an “average sample”. The pooled sample was then split into 4 aliquots, each 10 mL. Increasing concentrations of platinum were added (0, 1, 2, and 3 ng mL^-1^) to each of the aliquots.

- 1. **ICP-MS measurements of blood samples**

The matrix-matched standards and the digested serum samples were measured on an iCAP Qc quadrupole ICP-MS instrument (Thermo Fisher Scientific, Waltham, MA, USA). A glass cyclonic spray-chamber was used in combination with a quartz torch and quartz injector tube, as well as a Teflon® pneumatic nebulizer. Two isotopes of platinum (m/z = 194 and m/z = 195) and two isotopes of the internal standard (m/z = 113 and m/z = 115) were measured in ’kinetic energy discrimination’ mode (KED-mode). This mode allows the elimination of polyatomic spectral interferences which might impact the measurement.

- 1. **Quantification of serum samples**

Concentrations in the digested solutions were determined via matrix-matched calibration. These concentrations were then converted into ng mL^-1^ in the native serum sample, considering dilution factors which were applied during sample preparation. The response of the internal standard was constant over all samples and standards, suggesting that matrix-effects were negligible in the investigated samples. Similarly, both Pt isotopes resulted in the same quantitative result, suggesting that spectral and non-spectral interferences did not impact the results.

- 1. **LA-ICP-MS settings for tissue imaging**

The imaging experiments were performed with a 40-µm diameter laser beam. In the interest of complete but yet controlled ablation behavior the used laser energy and firing rate were adjusted to the analyzed materials in every experiment. The laser warmed up for 10 sec before firing the sample surface. For the ablation of the samples line scan patterns were used; adjoining lines while each line was ablated in the same direction. Before measurement, the sample chamber was purged with helium at a flow of 1 L min^-1^ for 30 min after the sample were placed. Two platinum isotopes were analyzed, the signal of ^195^Pt for data evaluation and the signal of the ^194^Pt isotope for data verification. These two isotopes always showed comparable patterns. As pseudo-internal standard, signal of ^197^Au was used, moreover, in order to correlate the obtained distribution information with medical knowledge and morphological structures, the intensities of the ^13^C, ^31^P and ^34^S isotopes were also recorded. Typical measurement settings of solid samples are summarized in Supplementary Materials Table 2. In each experiment, a sputtered gold layer for a maximum ^197^Au signal was used to optimize the MS instrumentation's tune settings.

**Supplementary Materials Table 2. Typical parameters of the solid sampling experiments.**

| Laser ablation system | New Wave 213 |
| --- | --- |
| Average fluence | 4.9 J cm^-2^ |
| Laser diameter | 10–100 mm |
| Scan speed | 25–100 mm s^-1^ |
| Repetition rate | 10 Hz |
| Carrier gas flow (He) | 1 L min^-1^ |
| Make-up gas flow (Ar) | 0.8 L min^-1^ |
| ICP-MS instrumentation | Thermo iCAP Q |
| Aux. gas flow | 0.8 L min^-1^ |
| Cool gas flow | 14 L min^-1^ |
| Dwell time per isotope | 10 msec |
| RF power | 1550 W |
| Cones | Ni |
| Mass resolution | m Δm^-1^ = 300 |
| Measured isotopes | ^13^C, ^31^P, ^34^S, ^194^Pt, ^195^Pt, ^197^Au |

- 1. **Tissue sample preparation for LA-ICP-MSI measurements**

Cryosections were prepared as described above and let dry at RT. A thin gold layer was then sputtered to the samples as pseudo-internal standard for LA-ICP-MSI analysis. This was performed by an Agar B7340 sputter coater (Agar Scientific Limited, Essex, UK) equipped with a gold sputtering target. Before every sputtering process a 4-cm distance was set between the sample and the gold target. To establish comparability of different analyses the time of metallization was also kept constant and was optimized in prior experiments. A sputtering time of 10 sec. was controlled by timer. The sample cell was evacuated to a pressure of 0.1 mbar and the sputtering current was adjusted to 10 mA.
