# Supplementary material for: Laser ablation‐inductively coupled plasma‐mass spectrometry analysis reveals differences in chemotherapeutic drug distribution in surgically resected pleural mesothelioma
Source: Br J Clin Pharmacol. 2023 Jul 14;89(11):3364–74. doi: 10.1111/bcp.15813 (PMC10952999; doi:10.1111/bcp.15813)
Supplement: Supplementary file 4 — Supporting information Table S1 Pt levels of full tissue specimens and serum according to patients' characteristics Supporting information Table S2 Vascularization and mean Pt levels of tumourous and nontumourous tissue areas (Mann‐Whitney test) Supporting information Table S3 Pt levels with regards to the type of administered chemotherapeutic agent: cisplatin vs carboplatin (Mann‐Whitney test) [file BCP-89-3364-s003.docx]

**Supplementary Tables**

Table 1. Pt levels of full tissue specimens and serum according to patients' characteristics.

|  | Number of patients (%) | Full tissue section mean Pt level  (μg g^-1^) | p value | Serum Pt concentration  (μg L^-1^) | p value |
| --- | --- | --- | --- | --- | --- |
| **All patients** | 25 (100%) |  |  |  |  |
| **Age (years)** |  |  |  |  |  |
| <64.5 | 12 (48%) | 0.81 | 0.289 | 239.1 | 0.201 |
| ≥64.5 | 13 (52%) | 1.25 |  | 173.7 |  |
| **Gender** |  |  |  |  |  |
| male | 19 (76%) | 1.05 | 0.589 | 200.8 | 0.824 |
| female | 6 (24%) | 1.01 |  | 218.7 |  |
| **Histology** |  |  |  |  |  |
| Epithelioid | 21 (84%) | 1.11 | 0.17 | 214.1 | 0.481 |
| Non-epithelioid | 4 (16%) | 0.66 |  | 158.1 |  |
| **IMIG pathol. stage** |  |  |  |  |  |
| Early (I/II) | 16 (64%) | 0.99 | 0.713 | 175.7 | 0.149 |
| Late (III/IV) | 9 (36%) | 1.14 |  | 257.3 |  |
| p values refer to full tissue section mean Pt levels and serum Pt concentrations between patient subgroups (Mann-Whitney test); Pt, platinum; PM, pleural mesothelioma; IMIG, International Mesothelioma Interest Group | | | | | |

Table 2. Vascularization and mean Pt levels of tumorous and non-tumorous tissue areas (Mann-Whitney test).

|  |  | MVA (%) | Mean Pt level  (µg g^-1^) | | Spearman r | p value |
| --- | --- | --- | --- | --- | --- | --- |
| n |  | 25 | 25 | | - | - |
| tumorous areas | mean±SEM | 3.66±0.61 | 0.83±0.15 | -0.122 | | 0.56 |
|  | median | 2.66 (0.37-11.38) | 0.51 (0.11-3.28) |  |  |  |
| n |  | 17 | 17 | | - | - |
| non-tumorous areas | mean±SEM | 3.78±0.6 | 1.21±0.16 | -0.222 | | 0.392 |
|  | median | 3.35 (0.56-11.09) | 1.05 (0.22-2.31) |  |  |  |
| MVA, Microvessel Area | | | | | | |

Table 3. Pt levels with regards to the type of administered chemotherapeutic agent; cisplatin vs. carboplatin (Mann-Whitney test).

|  |  | Cisplatin | Carboplatin | p value |
| --- | --- | --- | --- | --- |
| n |  | 16 | 7 | - |
| serum Pt concentration | mean±SEM | 223.3±30.6 µg L^-1^ | 163.9±38.6 µg L^-1^ | 0.3 |
|  | median | 199.5 (50.7-408.4) | 161.3 (70.6-367.3) |  |
| full tissue section mean Pt level | mean±SEM | 1.11±0.22 µg g^-1^ | 0.98±0.16 µg g^-1^ | 0.867 |
|  | median | 0.73 (0.2-3.28) | 0.83 (0.46-1.6) |  |
| tumorous tissue mean Pt level | mean±SEM | 0.9±0.23 µg g^-1^ | 0.8±0.14 µg g^-1^ | 0.442 |
|  | median | 0.48 (0.11-3.28) | 0.6 (0.44-1.32) |  |
| n |  | 13 | 5 | - |
| non-tumorous tissue mean Pt level | mean±SEM | 1.19±0.21 µg g^-1^ | 1.46±0.29 µg g^-1^ | 0.571 |
|  | median | 1.27 (0.22-2.31) | 1.12 (0.83-2.26) |  |
